# Evaluation of Humoral and Cellular Immune Responses to the SARS-CoV-2 Vaccine in Patients With Common Variable Immunodeficiency Phenotype and Patient Receiving B-Cell Depletion Therapy

**DOI:** 10.3389/fimmu.2022.895209

**Published:** 2022-04-29

**Authors:** Arnau Antolí, Gemma Rocamora-Blanch, Mario Framil, Virgínia Mas-Bosch, Sergio Navarro, Carla Bermudez, Sergio Martinez-Yelamos, Eva Dopico, Laura Calatayud, Nadia Garcia-Muñoz, Luis Humberto Hernández-Benítez, Antoni Riera-Mestre, Jordi Bas, Cristina Masuet-Aumatell, Raúl Rigo-Bonnin, Francisco Morandeira, Xavier Solanich

**Affiliations:** ^1^ Department of Internal Medicine, Hospital Universitari de Bellvitge, L’Hospitalet de Llobregat, Spain; ^2^ Bellvitge Biomedical Research Institute (IDIBELL), L’Hospitalet de Llobregat, Spain; ^3^ Adults Primary Immunodeficiency Unit (UFIPA), Hospital Universitari de Bellvitge, L’Hospitalet de Llobregat, Spain; ^4^ Department of Immunology, Hospital Universitari de Bellvitge, L’Hospitalet de Llobregat, Spain; ^5^ Department of Neurology, Hospital Universitari de Bellvitge, L’Hospitalet de Llobregat, Spain; ^6^ Faculty of Medicine and Health Sciences, Universitat de Barcelona, Barcelona, Spain; ^7^ Deparment of Microbiology, Hospital Universitari de Bellvitge, L’Hospitalet de Llobregat, Spain; ^8^ CIBER de Enfermedades Respiratorias, Instituto de Salud Carlos III, Madrid, Spain; ^9^ Blood Bank Department - Banc de Sang i Teixits (BST), Hospital Universitari de Bellvitge, L’Hospitalet de Llobregat, Spain; ^10^ Department of Preventive Medicine, Hospital Universitari de Bellvitge, L’Hospitalet de Llobregat, Spain; ^11^ Department of Clinical Laboratory, Hospital Universitari de Bellvitge, L’Hospitalet de Llobregat, Spain

**Keywords:** SARS-CoV-2, vaccine, COVID-19, multiple sclerosis, anti-CD20 therapies, CVID

## Abstract

**Introduction:**

SARS-CoV-2 vaccines’ effectiveness is not yet clearly known in immunocompromised patients. This study aims to assess the humoral and cellular specific immune response to SARS-CoV-2 vaccines and the predictors of poor response in patients with common variable immunodeficiency (CVID) phenotype and in patients treated with B-cell depletion therapies (BCDT), as well as the safety of these vaccines.

**Methods:**

From March to September 2021, we performed a prospective study of all adult patients who would receive the SARS-CoV-2 vaccination and were previously diagnosed with (i) a CVID syndrome (CVID phenotype group; n=28) or (ii) multiple sclerosis (MS) treated with B-cell depleting therapies three to six months before vaccination (BCD group; n=24). Participants with prior SARS-CoV-2 infection; or prior SARS-CoV-2 vaccine administration; or use of any immunosuppressant (except BCDT in MS group) were excluded. A group of subjects without any medical condition that confers immunosuppression and who met all study criteria was also assessed (control group; n=14). A chemiluminescence immunoassay was used to determine pre- and post-SARS-CoV-2 vaccine anti-S IgG antibodies. T-cell specific response was assessed by analysis of pre- and post-SARS-CoV-2 vaccination blood samples with an interferon-gamma release assay. The baseline blood sample also included several biochemical, haematological and immunological analyses.

**Results:**

SARS-CoV-2 vaccines are safe in immunocompromised patients, although their effectiveness was lower than in healthy individuals. CVID phenotype patients showed impaired humoral (29%) and cellular (29%) response, while BCD patients fundamentally presented humoral failure (54%). Low IgA values, low CD19+ peripheral B cells, low switched memory B cells, and a low CD4+/CD8+ ratio were predictors of inadequate specific antibody response in CVID phenotype patients. No factor was found to predict poor cellular response in CVID phenotype patients, nor a defective humoral or cellular response in BCD patients.

**Conclusion:**

The effectiveness of SARS-CoV-2 vaccines in CVID phenotype and BCD patients is lower than in healthy individuals. Knowledge of predictive factors of humoral and cellular response failure in immunocompromised patients could be very useful in clinical practice, and thus, studies in this regard are clearly needed.

## 1 Introduction

In December 2019, a novel coronavirus (SARS-CoV-2) causing an emerging disease (COVID-19) was first recognized in Wuhan (China) and spread globally (1, 2). In March 2022, the COVID-19 pandemic continued to spread worldwide, causing considerable morbidity and mortality, with almost 6 million deaths and more than 400 million confirmed cases (3).

Vaccines against SARS-CoV-2 were developed with the aim of protecting the population from infection, severe disease and mortality, becoming the principal tool for containing the pandemic. *in vivo.* Although SARS-CoV-2 vaccines provide robust protection in immunocompetent individuals, the immunogenicity of these vaccines in patients with immunosuppressant conditions is not well established (4–7).

Impaired immune response to the SARS-CoV-2 vaccines may be due to inborn errors of immunity (IEI). Common variable immunodeficiency (CVID) is the most frequent IEI disease with symptomatic primary antibody deficiency (8). Antibody production is impaired due to intrinsic molecular defects of B cells or defects that compromise B and T cell collaboration (9). Cellular immunity can be intact, although some cases present mild cellular defects (10–12). Therefore, humoral as well as cellular response to SARS-CoV-2 vaccination could be impaired in these patients.

SARS-CoV-2 vaccine response may also be impaired in secondary immunodeficiencies (SID) which can be caused by multiple factors that alter an intrinsically normal immune system. These factors include infectious agents such as the human immunodeficiency virus, medications, metabolic diseases and environmental conditions (13). Rituximab (RTX) and ocrelizumab (OCR) are anti-CD20 therapies widely used as treatments in B lymphocyte-mediated autoimmune diseases. B-cell depleting therapies (BCDT) can also affect antibody production (14, 15). It should be noted that the immune defects present in patients with SID can be driven by the disease or other treatments received to control its course. In addition, immunosuppression has a dynamic behaviour in the host over time. Accordingly, it has previously been shown that disease-modifying therapies decrease the immunogenicity of influenza, pneumococcal and tetanus vaccination (15, 16).

Some IEI and SID patients have a higher risk of severe COVID-19 disease (17, 18). SARS-CoV-2 vaccines authorised in Europe and the United States are considered safe in immunocompromised patients, but their effectiveness is not yet clearly known (19, 20). This study aims to assess the humoral and cellular specific immune response to SARS-CoV-2 vaccines and the predictors of poor response in patients with CVID phenotype and in patients treated with BCDT, as well as the safety of these vaccines.

## 2 Methods

### 2.1 Study Design and Patients

This study was conducted in a university hospital that is a referral centre for 2 million inhabitants with high-complexity diseases from the Southern area of Catalonia. From March to September 2021, we performed a prospective study of all patients who would receive the SARS-CoV-2 vaccination and met the following inclusion criteria and none of the exclusion criteria. The inclusion criteria were: (1) voluntary agreement to participate in the study and provide written informed consent; (2) age older than 18 years old; (3) IEI patients with CVID syndrome (CVID phenotype group), or multiple sclerosis (MS) patients treated with BCDT three to six months before vaccination (BCD group). The exclusion criteria were: (1) prior clinical or laboratory evidence of SARS-CoV-2 infection; (2) prior SARS-CoV-2 vaccine administration; (3) use of any immunosuppressant, except anti-CD20 therapies in the MS group. A group of subjects without any medical condition conferring immunosuppression, and who met all the study criteria was also evaluated to have reference values for our healthy population (control group). This last group contains approximately half of the patients as the other two groups since the humoral and cellular response to SARS-CoV-2 vaccines in healthy population has been extensively studied and is not the aim of this study.

The protocol was approved by the Ethics Committee of the Hospital Universitari de Bellvitge (Barcelona, Spain; approval number EOM009/21). All participants provided signed informed consent before the study procedures. This trial complies with the Declaration of Helsinki, Good Clinical Practice guidelines, and personal and clinical data were collected by the Spanish Data Protection Act (*Ley Orgánica 3/2018 de 5 de diciembre de Protección de Datos Personale*s).

### 2.2 Procedures

Before SARS-CoV-2 vaccination, serological and interferon-gamma release assay (IGRA) tests were performed to exclude previous asymptomatic SARS-CoV-2 infection. This baseline blood sample included different biochemical (albumin, creatinine, and C-reactive protein), haematological (leukocyte, lymphocyte, and absolute neutrophil counts), and immunological analyses (immunoglobulin isotypes, lymphocyte subpopulations, and complement factors C3, C4 and B). Biochemical analysis and immunoglobulins A, G and M levels were measured using a Cobas^®^ 8000 platform (Roche Diagnostics, Risch-Rotkreuz, Switzerland), haematological analyses were performed with a Sysmex^®^ XN2000 analyser (Sysmex Europe GmbH, Norderstedt, Germany), complement factors were determined using a Behring Nephelometer II System^®^ (Siemens Healthcare Diagnostics, Marburg, Germany), and lymphocyte subpopulations were analysed with the Navios EX flow cytometer^®^ (Beckman-Coulter, Brea, CA, USA). Isohaemagglutinins were analysed with an automated analyser, gel technique, Erytra Eflexis^®^ (Grifols, Barcelona, Spain).

Patients were vaccinated following the standard of care and vaccine availability. By the time this study was performed, four vaccines had already been approved by the European Medicines Agency (21) (two mRNA and two viral vector-based vaccines): BNT162b2 (Comirnaty^®^; BioNTech and Pfizer) (22, 23), mRNA-1273 (Spikevax^®^; Moderna) (24), ChAdOx1 nCoV-19 (Vaxzevria^®^; AstraZeneca) (25) and Ad26.COV2.S (COVID-19 Vaccine Janssen^®^; Johnson & Johnson) (26).

A second blood sample was obtained within a minimum period of 28 days after the second dose of SARS-CoV-2 vaccine administration, repeating serological and IGRA tests to evaluate humoral and cellular response. Additionally, some participants were voluntarily vaccinated against *Salmonella typhi* (Typhim Vi vaccine) to evaluate polysaccharide immune response.

### 2.3 SARS-CoV-2 Antibody Testing

To assess the humoral response to the SARS-CoV-2 vaccines, IgG antibodies against the spike subunit S1 (holds the spike protein receptor-binding domain) and S2 (transmembrane subunit) glycoprotein of the virus were measured by chemiluminescence immunoassays according to the manufacturer’s instructions (LIAISON^®^SARS-CoV-2 TrimericS IgG (DiaSorin S.p.A, Saluggia, Vercelli, Italy)). The test was performed in a LIAISON^®^ XL platform (DiaSorin). According to the manufacturers’ information, the antibody concentration was measured as binding antibody units (BAU)/mL following the WHO International Standard. The quantification range is between 4.81 and 2080 BAU/mL, with a positive cut-off being defined as ≥33.8 BAU/mL. The test was validated by the WHO International Standard for anti-SARS-CoV-2 immunoglobulin (NIBSC 20/136) (27).

To exclude a previous SARS-CoV-2 infection, antibodies against the nucleocapsid (anti-N) of the virus were determined by a qualitative chemiluminescence immunoassay, Elecsys^®^Anti-SARS-CoV-2 (Roche Diagnostics Penzberg, Germany) in a Cobas^®^ 8000 platform. This CE/IVD-marked assay is designed for detecting different antibody subclasses (IgM, IgA, and primarily IgG). The results are reported as the cut-off index (COI), and results with a COI ≥1.0 are interpreted as positive.

### 2.4 SARS-CoV-2 Interferon-Gamma Release Assay Test

According to the manufacturer’s instructions, T-cell specific response was assessed by analysis of pre- and post- vaccination peripheral whole blood samples with a commercial IGRA kit (Reference ET 2606-3003 and EQ 6841-9601, Euroimmun, Medizinische Labordiagnostika AG, Lübeck, Germany). In brief, 500 µL of fresh heparinized blood was incubated at 37°C for 20-24 h in three different tubes under different conditions: unstimulated, stimulated with peptides of the S1 domain of the SARS-CoV-2 spike protein and stimulated with a mitogen causing unspecific interferon-γ (IFN-γ) secretion. The latter tube was used to verify whether the sample contains immune cells with a sufficient ability to be activated. After the incubation, the stimulation tubes were centrifuged and 200 µL of plasma were harvested and stored at -80°C. IFN-γ concentration in the plasma fraction were measured by ELISA (Euroimmun) according to the manufacturer’s instructions. IFN-γ response was defined as the concentration of IFN-γ in the peptide stimulated tube minus the IFN-γ concentration in the unstimulated tube. According to the manufacturer’s instructions, values over 200 U/mL were considered positive.

### 2.5 *Salmonella typhi* Serological Test

Specific antibodies to the Typhim Vi vaccine were measured using the VaccZyme™ human Anti-S. typhi Vi IgG Enzyme Immunoassay kit (The Binding Site Group Ltd., Birmingham, UK). Antibodies from pre- and post- vaccination serum samples were measured following the manufacturer’s instructions. The results were expressed as U/mL (measurement range, 7.4–600 U/mL). The values of these responses are expressed as the ratio between pre- and post-immunization antibody levels. We used a three-fold increase between pre- and post- vaccination levels to define normal antibody response, and a post- vaccination concentration >32 U/mL according to a previous multicentre study in the Spanish population (28).

### 2.6 Statistical Analysis

Descriptive statistics were performed using frequency rates and percentages for categorical variables, while continuous variables were described as the median and interquartile range (IQR). Comparisons between groups were assessed using the Mann–Whitney U or Kruskal-Wallis tests for continuous variables. As appropriate, categorical comparisons were performed using the χ2 test or Fisher’s exact test. Statistical significance was defined as a *p*-value <0.05, and odds ratios (OR) and their 95% confidence intervals (CI) were also used for categorical variables.

A multivariable binary logistic regression analysis was performed to identify independent predictors that established a multivariable prediction model. To avoid multicollinearity, closely correlated variables (e.g., amount of CD3+ cells and percentage of CD3+ cells) were excluded before analysis. The Wald Forward Stepwise method was used to select the predictive variables considering a cut-off of 0.5, a maximum number of iterations of 30, and with stepwise probabilities of *p* < 0.05 (for entries) and *p* < 0.10 (for removals).

Analyses were performed using the statistical package SPSS version 25 (IBM Corp. Endicott, NY, USA).

## 3 Results

### 3.1 Patient Characteristics

**Figure 1:**
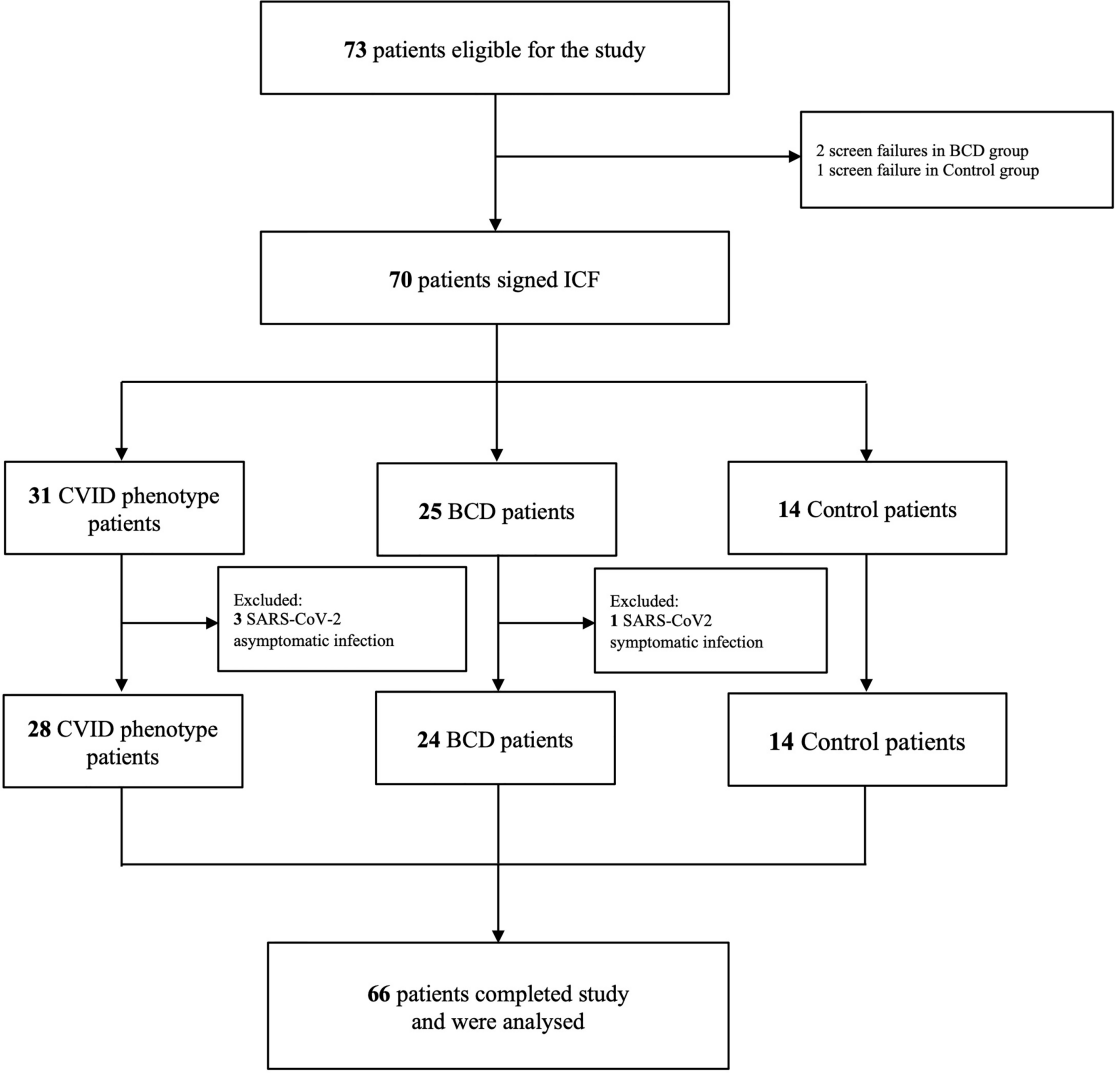
Study Flowchart. BCD, B-cell depleted; CVID, Common Variable Immunodeficiency; ICF, Informed Consent Form.

A total of 70 patients were prospectively recruited: 31 patients were included in the CVID phenotype group, 25 in the BCD group and 14 in the control group (**Figure 1**). Three patients in the CVID phenotype cohort were excluded because of previous asymptomatic SARS-CoV-2 infection determined by basal serology and SARS-CoV-2 IGRA positivity tests. One patient in the BCD cohort was excluded due to symptomatic SARS-CoV2 infection developed during the study. All the patients in the CVID phenotype cohort included in the analysis had a predominantly antibody deficiency: 24 CVID with an unknown genetic defect, one LRBA deficiency, one IKAROS haploinsufficiency and two patients with thymoma with hypogammaglobulinemia (Good syndrome). All the CVID phenotype patients fulfilled the European Society for Immunodeficiencies 2019 criteria for the diagnosis of CVID (29). Sixty-six patients completed the study and were included in the statistical analysis.

The main demographic, clinic and laboratory data are shown in **Table 1**. No significant differences were found in age or gender between groups (*p*=0.701 and *p*=0.800). Biochemical measurements did not show chronic kidney disease or hypoalbuminemia among the patients. There were no differences in terms of leucocytes (*p*=0.733), neutrophils (*p*=0.498) or lymphocytes (*p*=0.055). Baseline IgG levels were lower in CVID phenotype and BCD patients compared to controls. Twenty-three patients in the CVID phenotype group received immunoglobulin replacement therapy, whereas none of the BCD patients had received this treatment. IgA and IgM levels were significantly lower in the CVID phenotype group than in the BCD and control group (*p*<0.001). Immunoglobulin replacement therapy (IGRT) now contains SARS-CoV-2 antibodies (29–32) but in small amounts since no relevant differences in baseline anti-S or anti-N titers were observed between the CVID phenotype group (82% are treated with IGRT) and the other two groups (**Table 1**). In addition, 50% (*n*=14) of the patients in the CVID phenotype cohort had reduced CD3+CD4+CD45RA circulating naïve T lymphocytes. The median (IQR) blood count of circulating B cells (CD19+) was 0(0-0) x10^6^/L in SAD patients. In the BCD group, seven (29.2%) patients received RTX and 17 (70.8%) OCR. The median time (in days) between anti-CD20 treatment and the first dose of the SARS-CoV-2 vaccine was 118 days (IQR 96-139). The majority of the participants included in this study received the mRNA-1273 vaccine (PAD 85.7%; SAD 95.8%; Controls 92.9%).

**Table 1:** Main baseline demographic, clinical and laboratory data of the patients included in the study.

Variable	CVID phenotype (*n* = 28)	BCD (*n* = 24)	Control (*n* = 14)	*p*-value
**Demographics**				
Age, yr; median (IQR)	50 (41−68.5)	51 (46−56)	50.5 (30−65)	0.701
Sex (Male); *n* (%)	14 (50.0)	11 (45.8)	8 (57.1)	0.800
**Biochemical determinations**				
Albumin, g/L; median (IQR)	45.3 (42.5−47.5)	46.9 (44.0−47.6)	46.0 (46.0−48.0)	0.279
Creatinine, μmol/L; median (IQR)	71 (58−82.5)	58.5 (52−71.5)	70.5 (64−85)	0.094
C-reactive protein, mg/L; median (IQR)	2.43 (1.12−3.93)	1.31 (0.80−2.77)	0.71 (0.60−4.00)	0.075
**Haematological determinations**				
Leucocytes, x10^9^ cells/L; median (IQR)	6.09 (4.34−8.24)	6.56 (5.64−8.04)	6.60 (5.92−8.10)	0.733
Neutrophils, x10^9^ cells/L; median (IQR)	3.65 (2.79−5.07)	4.37 (3.48−5.23)	3.83 (3.08−5.38)	0.498
Lymphocytes, x10^9^ cells/L; median (IQR)	1.43 (1.03−2.39)	1.46 (1.11−1.78)	1.85 (1.64−2.12)	0.055
**Immunological determinations**				
Immunoglobulin G, mg/L; median (IQR)	8116 (6548−10146)	7836 (6759−9019)	11545 (10300−12315)	**<0.001**
Immunoglobulin M, mg/L; median (IQR)	207 (52.5−436)	609 (291−781)	921 (554−1383)	**<0.001**
Immunoglobulin A, mg/L; median (IQR)	50 (50−725)	1639 (1432−2503)	2204 (1882−2634)	**<0.001**
CD3+, x10^6^/L; median (IQR)	1063 (837−1837)	1208 (940−1474)	1413 (1184−1648)	0.273
CD3+, %; median (IQR)	81.0 (76.5−86.0)	87.5 (77.5−91.0)	81.0 (76.5−86.0)	**0.012**
CD3+CD4+, x10^6^/L; median (IQR)	602 (408−971)	778 (672−1133)	942 (785−1102)	**0.012**
CD3+CD4+, %; median (IQR)	42.0 (32.5−48.0)	58.5 (49.5−65.0)	51.0 (47.0−54.0)	**<0.001**
CD3+CD4+, < 200x10^6^/L; *n* (%)	1 (3.6)	0 (0.0)	0 (0.0)	0.507
CD3+CD4+CD45RA, < 10%; *n* (%)	14 (50.0)	2 (7.1)	3 (21.4)	**0.004**
CD3+CD8+; x10^6^/L, median (IQR)	411 (267−782)	281 (202−537)	436 (354−479)	0.266
CD3+CD8+; %, median (IQR)	31.0 (19.5−48.0)	23.0 (14.0−31.5)	21.5 (17.0−26.0)	**0.034**
(CD3+CD4+)/(CD3+CD8+) 1; median (IQR)	1.41 (0.73−2.46)	2.83 (1.49−4.11)	2.33 (1.94−2.79)	**0.007**
NK (CD3-CD16+CD56+), x10^6^/L; median (IQR)	118 (68.5−184)	217 (102−295)	203 (171−251)	**0.017**
NK (CD3-CD16+CD56+), %; median (IQR)	6.0 (4.0−14.0)	10.5 (7.0−22.0)	10.5 (8.0−13.0)	**0.027**
CD19+, x10^6^/L; median (IQR)	123 (40.5−215)	0 (0−0)	237 (195−262)	**<0.001**
CD19+, ≤ 1%; *n* (%)	5 (17.9)	24 (100.0)	0 (0.0)	**<0.001**
Switched memory B cells (CD19+CD27+IgD-IgM-), ≤ 2%; *n* (%)	15 (53.6)	24 (100.0)	0 (0.0)	**<0.001**
Transitional B cells (CD19+CD24hiCD27-CD38hi), < 9%; *n* (%)	25 (89.3)	24 (100.0)	0 (0.0)	0.272
Activated B cells (CD19+CD21loCD38lo), > 10%; *n* (%)	1 (3.6)	0 (0.0)	1 (7.1)	0.458
Absent haemagglutinins; *n* (%)	8 (29.6)	5 (21.7)	0 (0.0)	0.097
* Salmonella Typhi M* vaccine, deficient response; *n* (%)	25 (89.3)	18 (94.7)	0 (0.0)	**<0.001**
Complement C3, mg/L; median (IQR)	1225 (1070−1335)	1210 (1020−1415)	1165 (1090−1320)	0.963
Complement C4, mg/L; median (IQR)	257 (213−352)	260 (223−286)	230 (164−260)	0.152
Complement B, mg/L; median (IQR)	365 (316−424)	331 (300−423)	347 (274−377)	0.531
**SARS-CoV-2 tests**				
IgG anti-S, BAU/mL; median (IQR)	0.0 (0.0−1.40)	0.0 (0.0−0.0)	0.0 (0.0−0.0)	**0.025**
(IgG+IgA+IgM) anti-N, BAU/mL; median (IQR)	0.079 (0.071−1.22)	0.073 (0.069−0.075)	0.081 (0.074−0.088)	0.871
IGRA, U/mL; median (IQR)	5.45 (0.00−13.5)	0.53 (0.00−12.1)	2.00 (0.00−41.1)	0.893
**Treatments**				
Immunoglobulin Replacement Therapy, yes; *n* (%)	23 (82.1)	n.a.	n.a.	n.a.
Rituximab, yes; *n* (%)	n.a.	7 (29.2)	n.a.	n.a.
Ocrelizumab, yes; *n* (%)	n.a.	17 (70.8)	n.a.	n.a.
**Vaccines**				
BNT162b2; *n* (%)	3 (10.7)	0 (0)	1 (7.1)	0.192
mRNA-1273; *n* (%)	24 (85.7)	23 (95.8)	13 (92.9)	0.725
ChAdOx1 nCoV-19; *n* (%)	1 (3.6)	1 (4.2)	0 (0)	0.325
Ad26.COV2.S; *n* (%)	0 (0)	0 (0)	0 (0)	n.a.
**Others**				
Number of days between the antiCD20 therapy and the first vaccine administration; median (IQR)	n.a.	118 (96−139)	n.a.	n.a.
Number of days between the vaccine second dose and blood extraction for analysis; median (IQR)	28 (28−34)	29 (28−34)	33 (30−40)	0.235

IQR, interquartile range; n.a, not applicable; BCD, B-cell depleted; CVID, common variable immunodeficiency. Significant p-values are in bold.

### 3.2 Humoral Response

**Figure 2:**
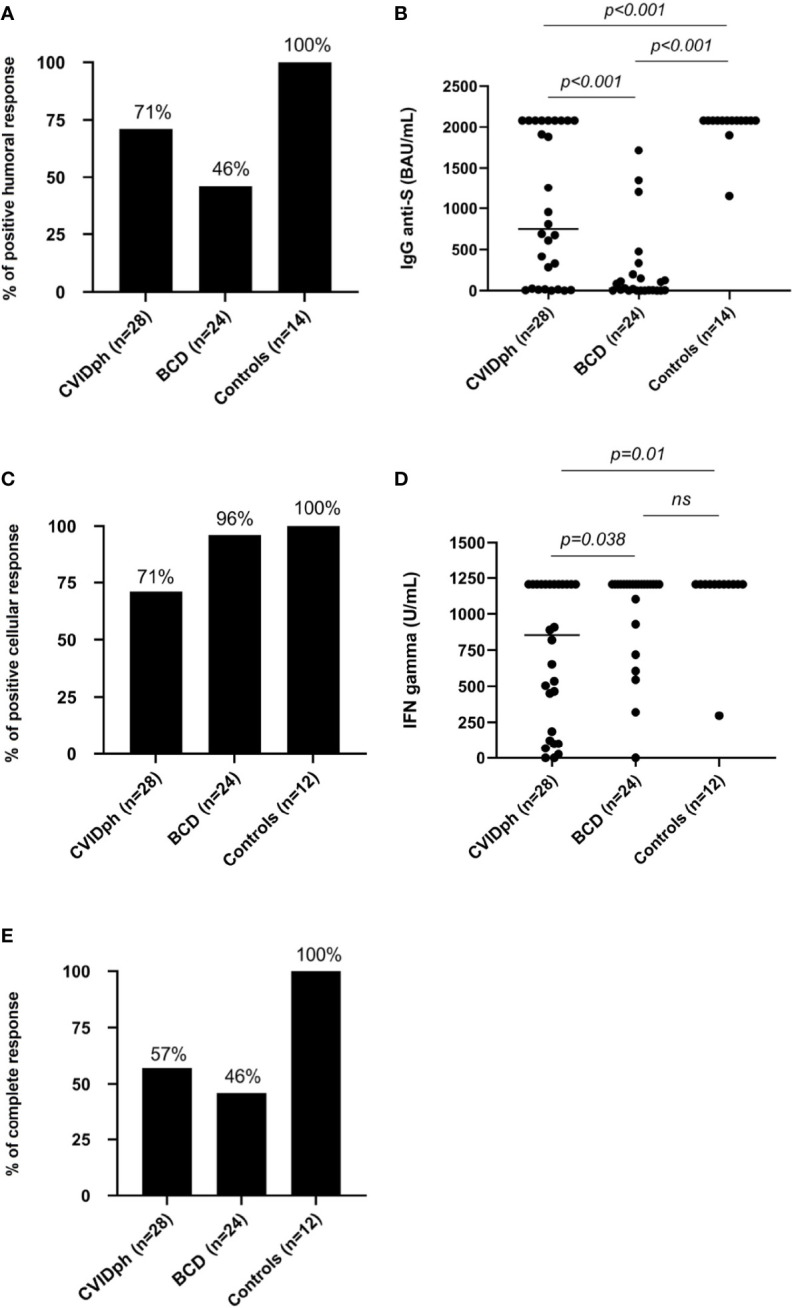
**(A)** Percentage of individuals with a positive humoral response using IgG anti-Spike protein SARS-CoV2 post-vaccination levels in the three groups (CVID phenotype, BCD and controls). **(B)** IgG anti-S levels post-vaccination using BAU in the three groups. **(C)** Percentage of individuals with a positive cellular response using IGRAs SARS-CoV-2 test post-vaccination in the three groups (CVID phenotype, BCD and controls). **(D)** IFN-γ concentration in U/mL post vaccination in the three groups. **(E)** Percentages of complete response (IgG anti-S and IGRA SARS-CoV-2 test) in the three groups (CVID phenotype, BCD and controls). BCD, B-cell depleted; CVID, Common Variable Immunodeficiency; CVIDph, Common Variable Immunodeficiency phenotype. ns, not significant.

Significant differences (*p*<0.001) in humoral response were found between the CVID phenotype, BCD and control groups (**Figures 2A, B**). All (100%) control group patients showed positive IgG anti-S levels (2080 BAU/mL; IQR 2080-2080). CVID phenotype patients showed lower IgG anti-S levels (1895 BAU/mL; IQR 677-2080) compared to the control group (*p*<0.001), achieving 71.4% of humoral response. None of the patients with Good syndrome or IEI with a known genetic defect (LRBA, IKAROS) presented IgG anti-S response. BCD patients presented the lowest IgG anti-S response (199 BAU/mL; IQR 114-1200), being significantly lower than controls (*p*<0.001) and CVID phenotype patients (*p*<0.001). Positive anti-S levels were only achieved in 46% of the BCD patients.

### 3.3 Cellular Response

**Figure 3:**
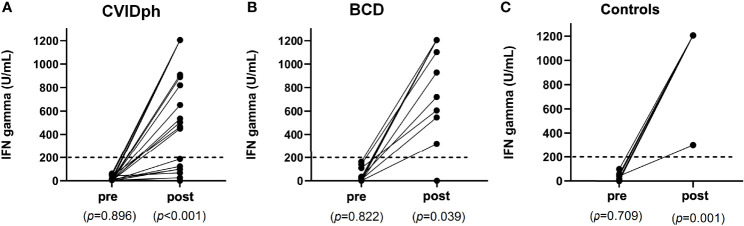
**(A)** Cellular response pre- and post-vaccination in CVID phenotype patients according to IFN-γ concentrations. **(B)** Cellular response pre- and post-vaccination in BCD patients according to IFN-γ concentrations. **(C)** Cellular response pre- and post-vaccination in control patients according to IFN-γ concentrations. BCD, B-cell depleted; CVID, Common Variable Immunodeficiency.

In relation to cellular response using a SARS-CoV-2 specific IGRA (**Figures 2C, D**), CVID phenotype patients had significantly lower IFN-γ levels than controls (855 U/mL; IQR 139-1205 vs. 1205 U/mL; IQR 1205-1205; *p*=0.011) and BCD patients (855 U/mL; IQR 139-1205 vs. 1205 U/mL; IQR 972-1205; *p*=0.038). CVID phenotype patients presented the lowest rate of cellular response (71%), while no significant statistical differences were observed in the cellular response rates between the control (100%) and BCD (96%) groups. IEI patients with a known genetic defect (LRBA, IKAROS) presented a cellular response, while only one of the two patients with Good syndrome had it. The control group showed a more homogeneous and robust IFN-γ response than the BCD group (**Figure 3**).

### 3.4 Complete (Humoral and Cellular) Response

Taking into account both humoral (IgG anti-S) and cellular (SARS-CoV2 IGRA test) immune response after receiving complete vaccination, BCD patients had the lowest percentage (46%) of respondents, followed by CVID phenotype patients (57%) (**Figure 2E**). This was mainly due to a worse humoral response in BCD patients, while the SARS-CoV-2 vaccines have been demonstrated to be highly effective in generating antibody and T-cell-specific responses in healthy populations.

### 3.5 Predictors of Poor Humoral and/or Cellular Response

#### 3.5.1 CVID Phenotype Group

**Figure 4:**
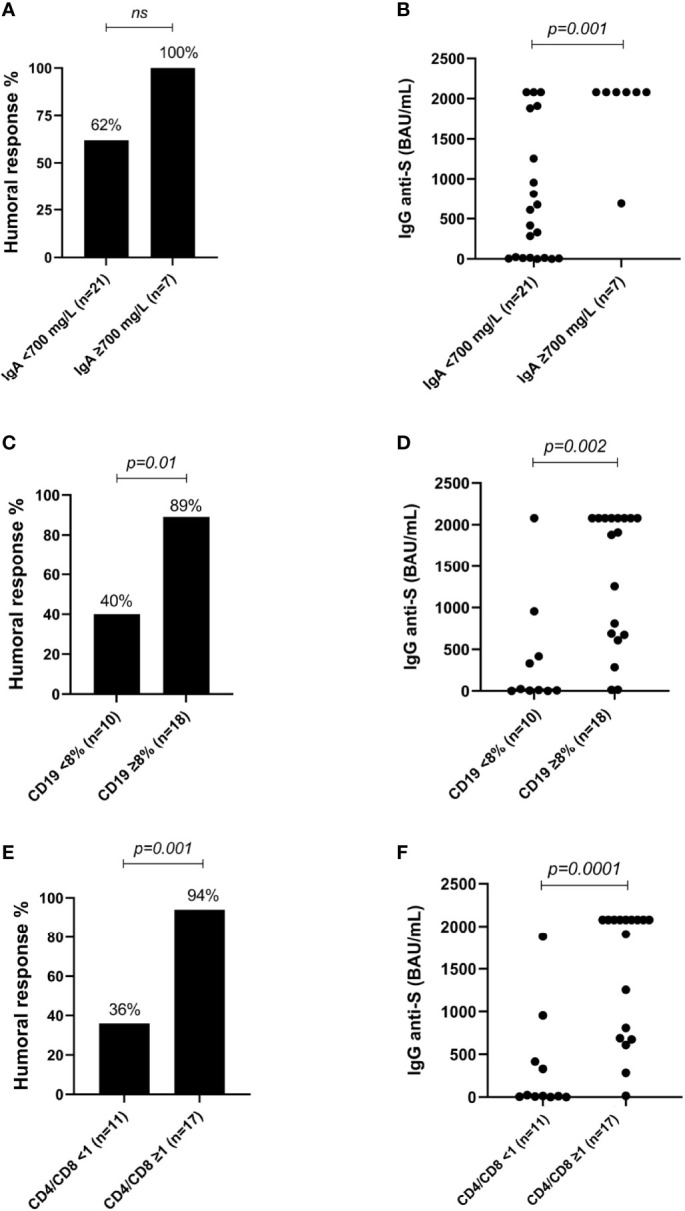
**(A)** Percentage of individuals with a positive humoral response depending on the IgA levels in patients with common variable immunodeficiency phenotype. **(B)** IgG anti-S levels depending on the IgA levels in patients with common variable immunodeficiency phenotype. **(C)** Percentage of individuals with a positive humoral response depending on the CD19+ cells in patients with common variable immunodeficiency phenotype. **(D)** IgG anti-S levels depending on the CD19+ cells in patients with common variable immunodeficiency phenotype. **(E)** Percentage of individuals with a positive humoral response depending on the CD4+/CD8+ ratio in patients with common variable immunodeficiency phenotype. **(F)** IgG anti-S levels depending on the CD4+/CD8+ ratio in patients with common variable immunodeficiency phenotype. ns, not significant.

Univariate analysis of CVID phenotype patients showed the following to be predictors of deficient humoral response: low IgA values (*p*=0.043), low CD19+ cells [cell count (*p*<0.001), percentage (*p*=0.008) or ≤1% (*p*<0.011)], switched memory B cells (CD19+CD27+IgD-IgM-) ≤2% (*p*=0.007), high %CD3+ cells (*p*=0.008) and low CD4+/CD8+ ratio (*p*=0.019) [due to low %CD3+CD4+ (*p*=0.033) as well as high %CD3+CD8+ (*p*=0.018)]. However, after the first iteration using the Wald Forward Stepwise method, multivariate analysis showed that none of these variables remained significant in the multivariate analysis (**Table 2**, **Figure 4** and **Supplementary Appendix Figure 1**), and no multivariate logistic regression equation could be obtained.

**Table 2:** Univariate and multivariate analysis of contributing factors to humoral immune response after vaccination in patients with common variable immunodeficiency phenotype.

Variable	Humoral immune response present (*n* = 20)	Deficient Humoral immune response (*n* = 8)	Univariate analysis		Multivariate analysis	
			OR	*p*	e^β^	*p*
Age, yr; median (IQR)	48.5 (39.5−66)	62.5 (45−72.5)	n.a.	0.165	n.i.	0.296
Lymphocytes, x10^9^ cells/L; median (IQR)	1.43 (1.04−2.52)	1.38 (0.78−1.87)	n.a.	0.469	n.i.	0.701
Albumin, g/L; median (IQR)	45.6 (43.8−47.4)	42.5 (39.0−47.8)	n.a.	0.281	n.i.	0.537
Creatinine, μmol/L; median (IQR)	71 (56−81.5)	67.5 (62.5−82.5)	n.a.	0.980	n.i.	0.997
Immunoglobulin G, mg/L; median (IQR)	8853 (6011−10146)	7496 (7150−10304)	n.a.	0.823	n.i.	0.716
Immunoglobulin M, mg/L; median (IQR)	294 (87−459)	71.5 (50−144)	n.a.	0.099	n.i.	0.214
Immunoglobulin A, mg/L; median (IQR)	135 (50−955)	50 (50−50)	n.a.	**0.043**	n.i.	0.560
CD3+, x10^6^/L; median (IQR)	1016 (847−1837)	1205 (663−1802)	n.a.	0.823	Ex.col.	Ex.col.
CD3+, %; median (IQR)	78.5 (72.5−82.5)	87.0 (81.0−93.5)	n.a.	**0.008**	n.i.	0.264
CD3+CD4+, x10^6^/L; median (IQR)	648 (458−1061)	424 (330−622)	n.a.	0.055	Ex.col.	Ex.col.
CD3+CD4+, %; median (IQR)	43.5 (36.5−50.0)	30.5 (25.0−37.5)	n.a.	**0.033**	n.i.	0.492
CD3+CD4+, < 200x10^6^/L; *n* (%)	0 (0.0)	1 (12.5)	n.a.	0.286	Ex.col.	Ex.col.
CD3+CD4+CD45RA, < 10%; *n* (%)	9 (45.0)	5 (62.5)	0.491 (0.091−2.636)	0.678	n.i.	0.390
CD3+CD8+, x10^6^/L; median (IQR)	385 (250−586)	642 (352−1228)	n.a.	0.237	Ex.col.	Ex.col.
CD3+CD8+, %; median (IQR)	26.5 (18.0−35.5)	50.5 (42.0−56.0)	n.a.	**0.018**	n.i.	0.350
(CD3+CD4+)/(CD3+CD8+), 1; median (IQR)	1.80 (1.07−2.66)	0.63 (0.46−0.82)	n.a.	**0.019**	Ex.col.	Ex.col.
NK (CD3-CD16+CD56+), x10^6^/L; median (IQR)	147 (75.5−196)	87.5 (23−133)	n.a.	0.199	n.i.	0.285
NK (CD3-CD16+CD56+), %; median (IQR)	7.0 (6.0−13.5)	5.0 (3.0−15.0)	n.a.	0.469	Ex.col.	Ex.col.
CD19+, x10^6^/L; median (IQR)	163 (86.7−267)	10 (0−51)	n.a.	**<0.001**	Ex.col.	Ex.col.
CD19+, %; median (IQR)	11.0 (8.0−13.5)	1.0 (0.0−7.0)	n.a.	**0.008**	Ex.col.	Ex.col.
CD19+, ≤ 1%; *n* (%)	0 (0.0)	6 (75.0)	n.a.	**<0.001**	n.i.	0.304
Switched memory B cells (CD19+CD27+IgD-IgM-), ≤ 2%; *n* (%)	7 (35.0)	8 (100.0)	n.a.	**0.007**	n.i.	0.607
Transitional B cells (CD19+CD24hiCD27-CD38hi), < 9%; *n* (%)	19 (95.0)	6 (75.0)	n.a.	0.385	n.i.	0.239
Activated B cells (CD19+CD21loCD38lo), > 10%; *n* (%)	1 (5.0)	0 (0.0)	n.a.	1.000	n.i.	0.992
Absent haemagglutinins; *n* (%)	5 (26.3)	3 (37.5)	0.595 (0.103−3.454)	0.658	n.i.	0.896
*Salmonella Typhi M* vaccine, deficient?? response; *n* (%)	17 (85.0)	8 (100.0)	n.a.	0.629	n.i.	0.986
Number of days between the vaccine second dose and blood extraction for analysis; median (IQR)	28 (28−33)	32.5 (28−40.5)	n.a.	0.566	n.i.	0.244
Immunoglobulin Replacement Therapy, yes; *n* (%)	15 (75.0)	8 (100.0)	n.a.	0.281	n.i.	0.604

IQR, interquartile range; OR, odds ratio; e^β^, multivariate odds ratio; n.a, not applicable; n.i., not included in the binary logistic regression equation; Ex.col., variable excluded in the multivariate analysis to avoid multicollinearity. Significant p-values are in bold.

None of the factors evaluated were found to be predictor of deficient cellular response (**Supplementary Appendix Table S1**).

While not being significant in the multivariate analysis, univariate analysis showed that the low number of CD19+ cells [cell count (*p*=0.001), percentage of CD19+ cells (*p*=0.013) or %CD19+ ≤ 1% (*p*=0.019)], and switched memory B cell (CD19+CD27+IgD-IgM-) values ≤ 2% (*p*=0.049) were associated with deficient complete humoral and cellular response (**Supplementary Appendix Table S2**).

#### 3.5.2 BCD Group

Regarding the BCD patients, no basal clinical or biological values were found to predict deficient humoral, cellular or complete immune responses in the univariate or multivariate analysis (**Supplementary Material Tables S3**–**S5**).

### 3.6 Safety

The SARS-CoV-2 vaccines were well tolerated, with no serious adverse events (AE) reported in any patient. A total of 142 self-reported mild AE were registered during the study period (**Supplementary Appendix Table S6**). The majority of these events (n=108) occurred after administration of the second vaccine dose. The most frequent AE (n=38) was pain/redness/swelling at the injection site. Most of the AE were of short duration (<72h) and requiring no treatment.

## 4 Discussion

Our data suggest that SARS-CoV-2 vaccines are safe in CVID phenotype and BCD patients, although impaired humoral and cellular immune response was shown compared to healthy controls. CVID phenotype patients showed impaired humoral (one third) and cellular (one third) immune response. Low IgA values, low CD19+ peripheral B cells, low switched memory B cells, and a low CD4+/CD8+ ratio were predictors of inadequate specific antibody response to SARS-CoV-2 vaccines in the CVID phenotype patients. None of these predictors was associated with deficient cellular response in CVID phenotype patients. Although a preserved cellular response was observed among BCD patients, more than half presented deficient humoral response. No factor in the univariate or multivariate analysis was found to predict deficient humoral or cellular response to the SARS-CoV-2 vaccine in BCD patients.

In the CVID phenotype cohort, an adequate humoral response was observed in 71% of the cases, similar to the ratios obtained in other IEI cohorts (69.2%-73.3%) (19, 33). Interestingly, our study is the first to predict which CVID phenotype patients might present a defective humoral response to SARS-CoV-2 vaccines. Based on our results, physicians should assess adequate humoral response following vaccination, especially in CVID phenotype patients presenting low IgA values, low CD19+ peripheral B cells, low switched memory B cells and a low CD4+/CD8+ ratio. The 71% cellular response using specific IGRAs in our SARS-CoV-2 vaccinated CVID phenotype patients is similar to the 73.1% previously reported in IEI patients using the ELIspot assay (19). Further studies involving cellular assays should be performed to investigate the T-cell specific response in CVID phenotype patients vaccinated against SARS-CoV-2. However, technologies to assess SARS-CoV-2 cellular responses *in vitro* are not available in most centres because they are complex, expensive, and time-consuming. On the other hand, classical delayed-type hypersensitivity response to the intradermal injection of a recombinant protein representative of the SARS-CoV-2 could be an alternative and easy method to evaluate the magnitude of anti-SARS-CoV-2 cellular responses *in vivo (* 34). Knowledge of predictive factors of cellular response failure to SARS-CoV-2 vaccination in CVID phenotype patients could be very useful in clinical practice if no cellular assays are available in our routine laboratory tests, and thus, studies in this regard are clearly needed.

BCDT represent a common factor for seroconversion failure (4, 7, 35–40). In our BCD patients, the ratio of seroconversion after full SARS-CoV-2 vaccination was lower (46% of IgG anti-S) than that described in recently published data in MS patients (36). Other studies in immune-mediated inflammatory diseases (IMID) patients treated with RTX reported 39-50% of seroconversion (37, 41), being percentages much closer to our results. It was of note, however, that despite presenting poor humoral response, 96% of our BCD patients achieved a cellular response. Other reports showed lower cellular responses (58%) in IMID patients under BCDT treatment using the ELISpot assay (37). More in-depth understanding of the role of B cells in T cell priming, differentiation and proliferation is necessary. Some studies have suggested that B cell antigen-presenting function facilitates T cell priming leading to concern about T cell response in B depleted patients (36). A recently published study has reported robust CD4 and CD8 T cell response to the SARS-CoV-2 vaccine in MS patients under BCDT (36) who also had selective defects in antigen-specific circulating T_FH_, preserved T_H_1 cell responses and augmented CD8 T cell responses. Although we did not detect predictors of poor humoral and/or cellular response after SARS-CoV-2 vaccination in MS patients under treatment with BCDT, several studies have suggested that deficient humoral response could be correlated with a lack of peripheral B cells (7, 36, 37, 42) and a short time between BCDT administration and SARS-CoV-2 vaccination (33, 36, 38).

While SARS-CoV-2 pandemic continues, the risks and benefits of BCDT administration must be assessed. The optimum time for SARS-CoV2 vaccination or further booster doses must be discussed with patients before starting immunosuppressant treatments. Knowledge of the humoral and cellular status of immunocompromised patients following receipt of the SARS-CoV-2 vaccine could help provide more precise and personalised preventive, clinical and therapeutic decisions (i.e. booster doses, early consult, early antiviral/monoclonal antibody treatment administration, among others). While a rapid vaccination policy was needed for the general population in the initial phases of the SARS-CoV2 pandemic, on entering an endemic phase, it is necessary to detect high-risk patients to avoid excessive vaccination and waste of resources.

Similar to what has previously been described in the literature, the SARS-CoV-2 vaccines were found to be safe in our immunosuppressed and healthy participants (4, 22–26, 43). No AEs events were reported in any of the three cohorts during the study period. The SARS-CoV-2 vaccines were well tolerated with non-long lasting mild side effects, indicating that they are safe as in other studies including an immunocompromised population (29, 34).

One strength of our study is that basal humoral and cellular assessment prior to vaccination, allowed excluding previously asymptomatic infected patients. Furthermore, none of the 66 participants assessed developed anti-N antibody positivity, so the asymptomatic infection during the study is reasonably excluded. Knowledge of the baseline patient characteristics provided predictors of poor vaccine response. Nevertheless, the study has several limitations. Firstly, our laboratory data were limited to the early post-vaccine period. The short-term follow-up did not allow evaluation of how this humoral and cellular immune response will protect the immunosuppressed patients against SARS-CoV-2 infection. Secondly, our results do not allow determining whether a third or even a fourth booster (44–46) dose generates stronger protection against SARS-CoV-2 infection. Thirdly, we did not evaluate antibody neutralising activity against SARS-CoV-2, although it appeared to correlate strongly with positive IgG anti-S antibody levels (47, 48). Fourthly, our results can only be extrapolated to the immunosuppressed patients assessed in the study. Finally, our MS patients received BCDT within three to six months prior to vaccination, making it difficult to evaluate if the humoral immune response would have been different if administered in a different interval.

In summary, SARS-CoV-2 vaccines were safe in immunocompromised patients, although their effectiveness was lower than in healthy individuals. CVID phenotype patients showed humoral and cellular defects, while BCD patients presented fundamentally humoral defects. Therefore, following complete vaccination it would be recommended to perform a SARS-CoV-2 serological test to evaluate the response of CVID phenotype and BCD patients as well as further cellular tests in CVID phenotype patients. On the other hand, some predictors of poor specific antibody response to the SARS-CoV-2 vaccine have been detected in CVID phenotype patients, which could be useful for decision making.

## Data Availability Statement

The raw data supporting the conclusions of this article will be made available by the authors without undue reservation.

## Ethics Statement

The studies involving human participants were reviewed and approved by Ethics Committee of the Hospital Universitari de Bellvitge (Barcelona, Spain; approval number EOM009/21). The patients/participants provided their written informed consent to participate in this study.

## Author Contributions

AA and GRB contributed equally to this work. RR-B, FM and XS designed the study. AA, GRB, RR-B, FM and XS input the study design. AA, GRB, SM-Y and XS assisted in patient management. MF, VM-B, CB, ED, LC, FM performed the serological, immunological analysis and interferon-gamma release assays. AA, GRB, RR-B and XS had full access to all data and took responsibility for the integrity and accuracy of the data. AA, GRB, RR-B, FM and XS drafted the manuscript. All authors revised and approved the final manuscript.

## Funding

With the support of the Departament de Salut de la Generalitat de Catalunya. We thank CERCA Programme/Generalitat de Catalunya for institutional support. HUB-IDIBELL investigators are supported by La Marató de TV3 Foundation (202115-30-31) and PECT-II-IDIBELL Internal Call for Innovation Projects.

## Conflict of Interest

The authors declare that the research was conducted in the absence of any commercial or financial relationships that could be construed as a potential conflict of interest.

## Publisher’s Note

All claims expressed in this article are solely those of the authors and do not necessarily represent those of their affiliated organizations, or those of the publisher, the editors and the reviewers. Any product that may be evaluated in this article, or claim that may be made by its manufacturer, is not guaranteed or endorsed by the publisher.

## Abbreviations

AE, Adverse event; BCD, B-cell depleted; BCDT, B-cell depleting therapies; CI, Confidence interval; CLL, Chronic lymphocytic leukaemia; COVID–19, Coronavirus Disease 2019; CVID, Common variable immunodeficiency; CVIDph, common variable immunodeficiency phenotype; ELISpot, Enzyme-linked immune absorbent spot; EMA, European Medicines Agency; HSCT, Haematological Stem Cell Transplant; HUB, Hospital Universitari de Bellvitge; IGRA, Interferon-gamma release assay; IMID, Immune-mediated inflammatory diseases; IEI, Inborn Errors of Immunity; IFN-γ, Interferon gamma; MERS-CoV, Middle East respiratory syndrome coronavirus; MS, Multiple sclerosis; OCR, Ocrelizumab; OR, Odds ratio; PCR, Polymerase chain reaction; PAD, Primary antibody deficiency; PID, Primary immunodeficiency; *r^2^*, *Coefficient of determination*; RTX, Rituximab; RNA, Ribonucleic acid; mRNA, Messenger ribonucleic acid; SAD, Secondary antibody deficiency; SARS-CoV, Severe acute respiratory syndrome coronavirus; SARS-CoV-2, Severe acute respiratory syndrome coronavirus 2; SID, Secondary immunodeficiency; WHO, World Health Organization.
