# Supplementary material for: Evaluation of Humoral and Cellular Immune Responses to the SARS-CoV-2 Vaccine in Patients With Common Variable Immunodeficiency Phenotype and Patient Receiving B-Cell Depletion Therapy
Source: Front Immunol. 2022 Apr 29;13:895209. doi: 10.3389/fimmu.2022.895209 (PMC9098934; doi:10.3389/fimmu.2022.895209)

Supplementary appendix Table S1. Univariate and multivariate analysis of factors contributing to cellular immune response after SARS-CoV-2 vaccination in patients with common variable immunodeficiency phenotype.

Variable	Cellular immune response present (*n*=20)	Deficient cellular immune response (*n*=8)	Univariate analysis		Multivariate analysis	
			OR	*p*	e^{β}	*p*
Age, yr; median (IQR)	48.5 (40.5–70.5)	61.5 (42–65)	n.a.	0.709	n.i.	0.547
Lymphocytes, x10^9 cells/L; median (IQR)	1.43 (1.07–2.44)	1.27 (0.90–2.00)	n.a.	0.354	n.i.	0.415
Albumin, g/L; median (IQR)	45.3 (42.5–48.2)	45.2 (42.5–46.2)	n.a.	0.784	n.i.	0.601
Creatinine, μmol/L; median (IQR)	70.5 (56–77)	77 (63–101)	n.a.	0.258	n.i.	0.062
Immunoglobulin G, mg/L; median (IQR)	8602 (6339–10743)	7498 (7004–9950)	n.a.	1.000	n.i.	0.794
Immunoglobulin M, mg/L; median (IQR)	207 (50–374)	257 (102–743)	n.a.	0.304	n.i.	0.106
Immunoglobulin A, mg/L; median (IQR)	50 (50–725)	68.5 (50–717)	n.a.	0.901	n.i.	0.952
CD3+, x10^6/L; median (IQR)	1063 (856–1940)	1098 (657–1699)	n.a.	0.438	Ex.col.	Ex.col.
CD3+, %; median (IQR)	79.5 (76.5–83.5)	85.0 (76.5–91.0)	n.a.	0.354	n.i.	0.185
CD3+CD4+, x10^6/L; median (IQR)	648 (467–971)	408 (349–920)	n.a.	0.304	n.i.	0.725
CD3+CD4+, %; median (IQR)	42.0 (32.0–46.5)	43.0 (32.5–53.0)	n.a.	0.533	Ex.col.	Ex.col.
CD3+CD4+, < 200x10^6/L; *n* (%)	1 (5.0)	0 (0.0)	n.a.	1.000	Ex.col.	Ex.col.
CD3+CD4+CD45RA, < 10%; *n* (%)	9 (45.0)	5 (62.5)	0.491 (0.091–2.636)	0.678	n.i.	0.472
CD3+CD8+, x10^6/L; median (IQR)	410 (267–861)	526 (240–782)	n.a.	1.000	Ex.col.	Ex.col.

CD3+CD8+, %; median (IQR)	30.5 (18.5–48.0)	31.0 (22.0–47.0)	n.a.	0.862	n.i.	0.866
(CD3+CD4+) / (CD3+CD8+), 1; median (IQR)	1.41 (0.64–2.44)	1.33 (0.73–2.46)	n.a.	1.000	Ex.col.	Ex.col.
NK (CD3-CD16+CD56+), x10^6/L; median (IQR)	150 (79–196)	85 (23–132)	n.a.	0.136	n.i.	0.351
NK (CD3-CD16+CD56+), %; median (IQR)	7.0 (6.0–13.5)	4.5 (3.0–14.5)	n.a.	0.354	Ex.col.	Ex.col.
CD19+, x10^6/L; median (IQR)	145 (40.5–266)	72 (39–153)	n.a.	0.258	Ex.col.	Ex.col.
CD19+, %; median (IQR)	10.5 (2.3–13.0)	8.0 (2.0–13.5)	n.a.	0.672	Ex.col.	Ex.col.
CD19+, ≤ 1%; *n* (%)	3 (15.0)	2 (25.0)	0.529 (0.070–3.978)	0.938	n.i.	0.574
Switched memory B cells (CD19+CD27+IgD-IgM-), ≤ 2%; *n* (%)	10 (50.0)	5 (62.5)	0.600 (0.112–3.214)	0.857	n.i.	0.637
Transitional B cells (CD19+CD24hiCD27-CD38hi), < 9 %; *n* (%)	19 (95.0)	6 (75.0)	6.333 (0.485–82.75)	0.385	n.i.	0.136
Activated B cells (CD19+CD21loCD38lo), > 10%; *n* (%)	0 (0.0)	1 (12.5)	n.a.	0.629	n.i.	0.116
Absent haemagglutinins; *n* (%)	6 (31.6)	2 (25.0)	1.385 (0.213–8.983)	1.000	n.i.	0.732
Salmonella Typhi M vaccine, deficient response; *n* (%)	17 (85.0)	7 (87.5)	0.810 (0.071–9.180)	1.000	n.i.	0.882
Number of days between the vaccine second dose and blood extraction for analysis; median (IQR)	28 (28–34)	30 (28–33.5)	n.a.	0.409	n.i.	0.527
Immunoglobulin Replacement Therapy, yes; *n* (%)	16 (80.0)	7 (87.5)	0.571 (0.054–6.079)	1.000	n.i.	0.601

IQR; interquartile range; OR, odds ratio; e^{β}, multivariate odds ratio; n.a, not applicable; n.i., not included in the binary logistic regression equation; Ex.col.; variable excluded in the multivariate analysis to avoid multicollinearity.

Supplementary appendix Table S2. Univariate and multivariate analysis of factors contributing to cellular and humoral immune response after SARS-CoV-2 vaccination in patients with common variable immunodeficiency phenotype.

Variable	Present Cellular and Humoral immune response (n=16)	Deficient Cellular or Humoral immune response (n=12)	Univariate analysis		Multivariate analysis	
			OR	p	e^{β}	p
Age, yr; median (IQR)	48.5 (39–69)	61.5 (42–67.5)	n.a.	0.371	n.i.	0.973
Lymphocytes, $x10^9$ cells/L; median (IQR)	1.43 (1.07–2.44)	1.38 (0.90–2.35)	n.a.	0.478	n.i.	0.823
Albumin, g/L; median (IQR)	45.3 (43.3–48.2)	45.2 (41.8–46.9)	n.a.	0.507	n.i.	0.234
Creatinine, μmol/L; median (IQR)	70.5 (56–77)	71 (62.5–84.5)	n.a.	0.599	n.i.	0.256
Immunoglobulin G, mg/L; median (IQR)	8853 (6011–10743)	7498 (7150–9950)	n.a.	0.873	n.i.	0.857
Immunoglobulin M, mg/L; median (IQR)	272 (59–436)	106 (50–393)	n.a.	0.631	n.i.	0.922
Immunoglobulin A, mg/L; median (IQR)	135 (50–909)	50 (50–272)	n.a.	0.302	n.i.	0.766
CD3+, $x10^6$/L; median (IQR)	1016 (856–1615)	1204 (656–2005)	n.a.	0.698	Ex.col.	Ex.col.
CD3+, %; median (IQR)	79.0 (72.5–82.5)	83.0 (78.0–91.0)	n.a.	0.110	n.i.	0.393
CD3+CD4+, $x10^6$/L; median (IQR)	648 (495–996)	443 (349–680)	n.a.	0.159	n.i.	0.790
CD3+CD4+, %; median (IQR)	42.5 (36.5–47.5)	35.5 (28.5–50.0)	n.a.	0.324	Ex.col.	Ex.col.
CD3+CD4+, < $200x10^6$/L; n (%)	0 (0.0)	1 (8.3)	n.a.	0.883	Ex.col.	Ex.col.
CD3+CD4+CD45RA, < 10%; n (%)	7 (58.3)	7 (43.8)	1.800 (0.396–8.182)	0.445	n.i.	0.890
CD3+CD8+, $x10^6$/L; median (IQR)	385 (250–510)	563 (300–946)	n.a.	0.347	Ex.col.	Ex.col.

CD3+CD8+, %; median (IQR)	25.0 (16.5–40.5)	42.0 (27.5–51.0)	n.a.	0.100	n.i.	0.958
(CD3+CD4+) / (CD3+CD8+), 1; median (IQR)	1.80 (0.97–2.69)	0.82 (0.52–1.84)	n.a.	0.114	Ex.col.	Ex.col.
NK (CD3-CD16+CD56+), x10^6/L; median (IQR)	150 (68.5–196)	90 (54.6–159)	n.a.	0.423	n.i.	0.479
NK (CD3-CD16+CD56+), %; median (IQR)	6.0 (6.0–12.5)	10.0 (3.0–15.5)	n.a.	0.873	Ex.col.	Ex.col.
CD19+, x10^6/L; median (IQR)	184 (105–297)	47.4 (2.7–108)	n.a.	**0.001**	Ex.col.	Ex.col.
CD19+, %; median (IQR)	11.0 (9.0–13.5)	2.0 (0.5–10.5)	n.a.	**0.013**	Ex.col.	Ex.col.
CD19+, ≤ 1%; *n* (%)	0 (0.0)	5 (41.7)	n.a.	**0.019**	n.i.	1.000
Switched memory B cells (CD19+CD27+IgD-IgM-), ≤ 2%; *n* (%)	6 (37.5)	9 (75.0)	0.200 (0.038–1.044)	**0.049**	n.i.	0.452
Transitional B cells (CD19+CD24hiCD27-CD38hi), < 9 %; *n* (%)	15 (93.8)	10 (83.3)	3.003 (0.239–37.04)	0.791	n.i.	0.163
Activated B cells (CD19+CD21loCD38lo), > 10%; *n* (%)	0 (0.0)	1 (8.3)	n.a.	0.883	n.i.	0.134
Absent haemagglutinins; *n* (%)	4 (26.7)	4 (33.3)	0.881 (0.139–3.817)	1.000	n.i.	0.448
Salmonella Typhi M vaccine, deficient response; *n* (%)	14 (87.5)	11 (91.7)	0.635 (0.051–7.937)	1.000	n.i.	0.952
Number of days between the vaccine second dose and blood extraction for analysis; median (IQR)	28 (28–34)	29 (28–33)	n.a.	0.664	n.i.	0.495
Immunoglobulin Replacement Therapy, yes; *n* (%)	11 (68.8)	11 (91.7)	0.200 (0.020–2.004)	0.319	n.i.	0.519

IQR, interquartile range; OR, odds ratio; eβ, multivariate odds ratio; n.a, not applicable; n.i., not included in the binary logistic regression equation; Ex.col.; variable excluded in the multivariate analysis to avoid multicollinearity.

Supplementary appendix Table S3. Univariate and multivariate analysis of factors contributing to humoral immune response after SARS-CoV-2 vaccination in patients with B-cell depleting therapy.

Variable	Present Humoral immune response (*n*=11)	Humoral immune response deficient (*n*=13)	Univariate analysis		Multivariate analysis	
			OR	*p*	e^{β}	*p*
Age, yr; median (IQR)	47 (44–55)	52 (47–56)	n.a.	0.277	n.i.	0.113
Lymphocytes, x10^9 cells/L; median (IQR)	1.42 (1.16–1.65)	1.57 (1.13–1.99)	n.a.	0.531	n.i.	0.161
Albumin, g/L; median (IQR)	46.4 (44.0–47.1)	47.1 (44.3–48.1)	n.a.	0.207	n.i.	0.434
Creatinine, μmol/L; median (IQR)	59 (54–74)	58 (53–67)	n.a.	0.608	n.i.	0.810
Immunoglobulin G, mg/L; median (IQR)	7283 (6275–9383)	7849 (7253–8522)	n.a.	1.000	n.i.	0.970
Immunoglobulin M, mg/L; median (IQR)	662 (349–971)	608 (231–706)	n.a.	0.277	n.i.	0.176
Immunoglobulin A, mg/L; median (IQR)	1731 (1563–2023)	1478 (1398–3154)	n.a.	0.569	n.i.	0.485
CD3+, x10^6/L; median (IQR)	1132 (902–1269)	1386 (974–1518)	n.a.	0.331	n.i.	0.216
CD3+, %; median (IQR)	88.0 (77.5–91.0)	87.0 (79.0–91.0)	n.a.	1.000	Ex.col.	Ex.col.
CD3+CD4+, x10^6/L; median (IQR)	733 (683–928)	816 (689–1274)	n.a.	0.649	Ex.col.	Ex.col.
CD3+CD4+, %; median (IQR)	61.0 (54.0–65.5)	54.0 (49.0–64.0)	n.a.	0.424	n.i.	0.154
CD3+CD4+, < 200x10^6/L; *n* (%)	0 (0.0)	0 (0.0)	n.a.	n.a.	Ex.col.	Ex.col.
CD3+CD4+CD45RA, < 10%; *n* (%)	1 (7.7)	1 (9.1)	1.000 (0.055–18.09)	1.000	n.i.	0.467
CD3+CD8+, x10^6/L; median (IQR)	276 (179–482)	339 (213–557)	n.a.	0.303	Ex.col.	Ex.col.

CD3+CD8+, %; median (IQR)	20.0 (14.0–27.0)	59 (54–74)	n.a.	0.361	n.i.	0.181
(CD3+CD4+) / (CD3+CD8+), 1; median (IQR)	2.95 (2.00–3.93)	2.03 (1.46–3.25)	n.a.	0.339	Ex.col.	Ex.col.
NK (CD3-CD16+CD56+), x10^6/L; median (IQR)	204 (92.1–271)	230 (116–300)	n.a.	0.776	n.i.	0.276
NK (CD3-CD16+CD56+), %; median (IQR)	10.0 (6.5–22.0)	11.0 (7.0–19.0)	n.a.	0.955	Ex.col.	Ex.col.
CD19+, x10^6/L; median (IQR)	0 (0.0)	0 (0.0)	n.a.	1.000	Ex.col.	Ex.col.
CD19+, %; median (IQR)	0.0 (0.0–0.0)	0.0 (0.0–0.0)	n.a.	1.000	Ex.col.	Ex.col.
CD19+, ≤ 1%; *n* (%)	11 (100.0)	13 (100.0)	n.a.	n.a.	n.a	n.a
Switched memory B cells (CD19+CD27+IgD-IgM-), ≤ 2%; *n* (%)	11 (100.0)	13 (100.0)	n.a.	n.a.	n.a	n.a
Transitional B cells (CD19+CD24hiCD27-CD38hi), < 9 %; *n* (%)	11 (100.0)	13 (100.0)	n.a.	n.a.	n.a	n.a
Activated B cells (CD19+CD21loCD38lo), > 10 %; *n* (%)	0 (0.0)	0 (0.0)	n.a.	n.a.	n.a	n.a
Absent haemagglutinins; *n* (%)	4 (36.4)	1 (8.3)	6.286 (0.577–68.42)	0.262	n.i.	0.596
Salmonella Typhi M vaccine, deficient response; *n* (%)	5 (83.3)	13 (100.0)	n.a.	0.684	n.i.	0.146
Number of days between the vaccine second dose and blood extraction for analysis; median (IQR)	30 (29–33.5)	29 (28–34)	n.a.	0.874	n.i.	0.692
Number of days between the rituximab/ocrelizumab therapy and the first dose of the vaccine; median (IQR)	116 (92–125)	121 (95–149)	n.a.	0.681	n.i.	0.405
Rituximab, yes; *n* (%)	2 (18.2)	5 (38.5)	0.356 (0.053–2.368)	0.523	n.i.	0.732

Ocrelizumab, yes; *n* (%)	9 (81.8)	8 (61.5)	2.813 (0.422−18.74)	0.523	n.i.	0.732

IQR, interquartile range; OR, odds ratio; e^{β}, multivariate odds ratio; n.a, not applicable; n.i., not included in the binary logistic regression equation; Ex.col.; variable excluded in the multivariate analysis to avoid multicollinearity.

Supplementary appendix Table S4. Univariate and multivariate analysis of factors contributing to cellular immune response after SARS-CoV-2 vaccination in patients with B-cell depleting therapy.

Variable	Cellular immune response present (*n*=23)	Deficient cellular immune response (*n*=1)	Univariate analysis		Multivariate analysis	
			OR	*p*	e^{β}	*P*
Age, yr; median (IQR)	50 (46–55.5)	59	n.a.	0.333	n.i.	0.345
Lymphocytes, x10^9 cells/L; median (IQR)	1.48 (1.18–1.78)	1.07	n.a.	0.417	n.i.	0.485
Albumin, g/L; median (IQR)	46.8 (44.0–47.6)	47.4	n.a.	0.583	n.i.	0.437
Creatinine, µmol/L; median (IQR)	59 (54.5–71.5)	47	n.a.	0.167	n.i.	0.299
Immunoglobulin G, mg/L; median (IQR)	7822 (6759–9019)	7849	n.a.	1.000	n.i.	0.921
Immunoglobulin M, mg/L; median (IQR)	609 (291–781)	436	n.a.	0.833	n.i.	0.649
Immunoglobulin A, mg/L; median (IQR)	1674 (1432–2503)	1603	n.a.	1.000	n.i.	0.851
CD3+, x10^6/L; median (IQR)	1265 (940–1474)	974	n.a.	0.667	Ex.col.	Ex.col.
CD3+, %; median (IQR)	87.0 (291–781)	91.0	n.a.	0.500	n.i.	0.586
CD3+CD4+, x10^6/L; median (IQR)	787 (691–1133)	514	n.a.	0.333	n.i.	0.241
CD3+CD4+, %; median (IQR)	60.0 (50.5–65.0)	48.0	n.a.	0.417	Ex.col.	Ex.col.
CD3+CD4+, < 200x10^6/L; *n* (%)	0 (0.0)	0 (0.0)	n.a.	n.a.	Ex.col.	Ex.col.
CD3+CD4+CD45RA, < 10%; *n* (%)	2 (8.7)	0 (0.0)	n.a.	1.000	n.i.	0.803
CD3+CD8+, x10^6/L; median (IQR)	279 (202–537)	417	n.a.	0.833	Ex.col.	Ex.col.
CD3+CD8+, %; median (IQR)	23.0 (14.0–30.5)	39.0	n.a.	0.083	n.i.	0.115

(CD3+CD4+) / (CD3+CD8+), 1; median (IQR)	2.85 (1.63–3.93)	1.23	n.a.	0.129	Ex.col.	Ex.col.
NK (CD3-CD16+CD56+), x10^6/L; median (IQR)	230 (113–295)	75	n.a.	0.417	n.i.	0.328
NK (CD3-CD16+CD56+), %; median (IQR)	11.0 (7.0–22.0)	7.0	n.a.	0.583	Ex.col.	Ex.col.
CD19+, x10^6/L; median (IQR)	0 (0–0)	0 (0.0)	n.a.	1.000	Ex.col.	Ex.col.
CD19+, %; median (IQR)	0.0 (0.0–0.0)	0.0	n.a.	1.000	Ex.col.	Ex.col.
CD19+, ≤ 1%; *n* (%)	23 (100.0)	1 (100.0)	n.a.	n.a.	n.a	n.a
Switched memory B cells (CD19+CD27+IgD-IgM-), ≤ 2%; *n* (%)	23 (100.0)	1 (100.0)	n.a.	n.a.	n.a	n.a
Transitional B cells (CD19+CD24hiCD27-CD38hi), < 9 %; *n* (%)	23 (100.0)	1 (100.0)	n.a.	n.a.	n.a	n.a
Activated B cells (CD19+CD21loCD38lo), > 10%; *n* (%)	0 (0.0)	0 (0.0)	n.a.	n.a.	n.a	n.a
Absent haemagglutinins; *n* (%)	5 (22.7)	0 (0.0)	n.a.	1.000	n.i.	0.716
Salmonella Typhi M vaccine, deficient response; *n* (%)	17 (94.4)	1 (100.0)	n.a.	1.000	n.i.	0.803
Number of days between the vaccine second dose and blood extraction for analysis; median (IQR)	29 (28–33.5)	34	n.a.	0.833	n.i.	0.556
Number of days between the rituximab/ocrelizumab therapy and the first dose of the vaccine; median (IQR)	118 (96–139)	114	n.a.	0.875	n.i.	0.699
Rituximab, yes; *n* (%)	6 (26.1)	1 (100.0)	n.a.	0.640	n.i.	0.197
Ocrelizumab, yes; *n* (%)	17 (73.9)	0 (0.0)	n.a.	0.640	n.i.	0.197

IQR, interquartile range; OR, odds ratio; e^{β}, multivariate odds ratio; n.a, not applicable; n.i., not included in the binary logistic regression equation; Ex.col.; variable excluded in the multivariate analysis to avoid multicollinearity.

Supplementary appendix Table S5. Univariate and multivariate analysis of factors contributing to cellular and humoral immune response after SARS-CoV-2 vaccination in patients with B-cell depleting therapy.

**Variable**	**Present Cellular and Humoral immune response (*n*=11)**	**Deficient Cellular or Humoral immune response (*n*=13)**	**Univariate analysis**		**Multivariate analysis**	
			**OR**	***p***	**e^{β}**	***p***
Age, yr; median (IQR)	47 (44–55)	52 (47–56)	n.a.	0.277	n.i.	0.113
Lymphocytes, x10^9 cells/L; median (IQR)	1.42 (1.16–1.65)	1.57 (1.13–1.99)	n.a.	0.531	n.i.	0.161
Albumin, g/L; median (IQR)	46.4 (44.0–47.1)	46.4 (44.0–48.1)	n.a.	0.207	n.i.	0.434
Creatinine, µmol/L; median (IQR)	59 (54–74)	58 (53–67)	n.a.	0.608	n.i.	0.810
Immunoglobulin G, mg/L; median (IQR)	7283 (6275–9383)	7849 (7253–8522)	n.a.	1.000	n.i.	0.970
Immunoglobulin M, mg/L; median (IQR)	662 (349–971)	608 (231–706)	n.a.	0.277	n.i.	0.176
Immunoglobulin A, mg/L; median (IQR)	1731 (1563–2023)	1478 (1397–3154)	n.a.	0.569	n.i.	0.485
CD3+, x10^6/L; median (IQR)	1132 (902–1269)	1386 (974–1518)	n.a.	0.331	n.i.	0.216
CD3+, %; median (IQR)	88.0 (77.5–91.0)	87.0 (79.0–91.0)	n.a.	1.000	Ex.col.	Ex.col.
CD3+CD4+, x10^6/L; median (IQR)	733 (683–928)	816 (689–1274)	n.a.	0.649	Ex.col.	Ex.col.
CD3+CD4+, %; median (IQR)	61.0 (54.0–65.5)	54.0 (49.0–64.0)	n.a.	0.424	n.i.	0.154
CD3+CD4+, < 200x10^6/L; *n* (%)	0 (0.0)	0 (0.0)	n.a.	n.a.	Ex.col.	Ex.col.
CD3+CD4+CD45RA, < 10%; *n* (%)	1 (9.1)	1 (7.7)	1.200 (0.066–21.74)	1.000	n.i.	0.467
CD3+CD8+, x10^6/L; median (IQR)	276 (179–482)	339 (213–557)	n.a.	0.303	n.i.	0.181

CD3+CD8+, %; median (IQR)	20.0 (14.0–27.0)	28.0 (16.0–32.0)	n.a.	0.361	Ex.col.	Ex.col.
(CD3+CD4+) / (CD3+CD8+), 1; median (IQR)	2.03 (1.46–3.25)	2.95 (2.43–3.93)	n.a.	0.339	Ex.col.	Ex.col.
NK (CD3-CD16+CD56+), x10^6/L; median (IQR)	204 (92–271)	230 (116–300)	n.a.	0.776	n.i.	0.276
NK (CD3-CD16+CD56+), %; median (IQR)	10.0 (6.5–22.0)	11.0 (7.0–19.0)	n.a.	0.955	Ex.col.	Ex.col.
CD19+, x10^6/L; median (IQR)	0 (0–0)	0 (0.0)	n.a.	1.000	Ex.col.	Ex.col.
CD19+, %; median (IQR)	0.0 (0.0–0.0)	0.0 (0.0–0.0)	n.a.	1.000	Ex.col.	Ex.col.
CD19+, ≤ 1%; *n* (%)	11 (100.0)	13 (100.0)	n.a.	n.a.	n.a	n.a
Switched memory B cells (CD19+CD27+IgD-IgM-), ≤ 2%; *n* (%)	11 (100.0)	13 (100.0)	n.a.	n.a.	n.a	n.a
Transitional B cells (CD19+CD24hiCD27-CD38hi), < 9 %; *n* (%)	11 (100.0)	13 (100.0)	n.a.	n.a.	n.a	n.a
Activated B cells (CD19+CD21loCD38lo), > 10%; *n* (%)	0 (0.0)	0 (0.0)	n.a.	n.a.	n.a	n.a
Absent haemagglutinins; *n* (%)	4 (36.4)	1 (8.3)	6.289 (0.577–66.66)	0.262	n.i.	0.596
Salmonella Typhi M vaccine, deficient response; *n* (%)	5 (83.3)	13 (100.0)	n.a.	0.684	n.i.	0.146
Number of days between the vaccine second dose and blood extraction for analysis; median (IQR)	30 (29–33.5)	29 (28–34)	n.a.	0.691	n.i.	0.692
Number of days between the rituximab/ocrelizumab therapy and the first dose of the vaccine; median (IQR)	115 (97–125)	122 (96–147)	n.a.	0.705	n.i.	0.689
Rituximab, yes; *n* (%)	2 (18.2)	5 (38.5)	0.355 (0.053–2.369)	0.523	n.i.	0.732

Ocrelizumab, yes; *n* (%)	9 (91.8)	8 (61.5)	2.809 (0.422−18.87)	0.523	n.i.	0.732

IQR, interquartile range; OR, odds ratio; e^{β}, multivariate odds ratio; n.a, not applicable; n.i., not included in the binary logistic regression equation; Ex.col.; variable excluded in the multivariate analysis to avoid multicollinearity.

Supplementary appendix Table S6. Adverse events related to SARS-CoV-2 vaccine administration.

Total Adverse Events	Number	Adverse Events After 1st Dose	Number	Adverse Events After 2nd Dose	Number
Pain/redness/swelling at the injection site	38	Pain/redness/swelling at the injection site	17	Pain/redness/swelling at the injection site	21
General discomfort	30	General discomfort	5	General discomfort	25
Fever	21	Fever	1	Fever	20
Arthromyalgia	10	Arthromyalgia	3	Arthromyalgia	7
Headache	8	Headache	2	Headache	6
Low-grade fever	8	Low-grade fever	0	Low-grade fever	8
Asthenia/weakness	8	Asthenia/weakness	0	Asthenia/weakness	8
Dysthermia	5	Dysthermia	2	Dysthermia	3
Shortness of breath	2	Shortness of breath	1	Shortness of breath	1
Cough	2	Cough	1	Cough	1
Paraesthesia	2	Paraesthesia	1	Paraesthesia	1
Sickness/Nausea	2	Sickness/Nausea	0	Sickness/Nausea	2
Chills	2	Chills	0	Chills	2
Temporal worsening of neurological focality	2	Temporal worsening of neurological focality	0	Temporal worsening of neurological focality	2
Dizziness	1	Dizziness	1	Dizziness	0
Itching/pruritus	1	Itching/pruritus	0	Itching/pruritus	1
Total AE Number	142	Total AE after 1st Dose	34	Total AE After 2nd Dose	108

AE, adverse event.

Supplementary figure 1. IgG anti-S titers (A) and percentage of humoral response (B) depending on the CD3+ cells in patients with common variable immunodeficiency phenotype.

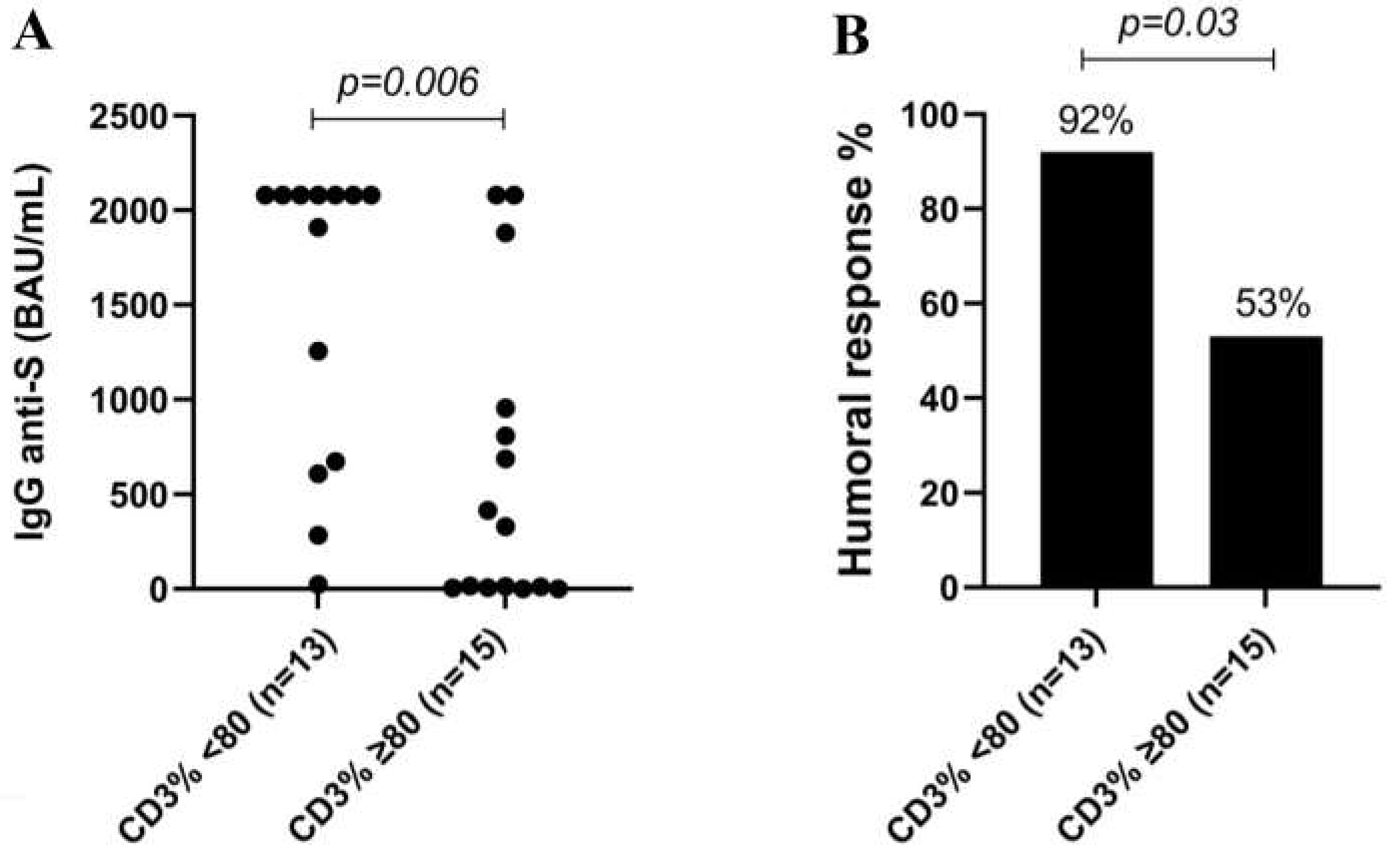

Supplement: Supplementary file 1 [file DataSheet_1.pdf]
